# Role of the Fungal Serological Marker Galactomannan Antigen in the Diagnosis of Invasive Fungal Infection: A Three-Year Retrospective Study

**DOI:** 10.7759/cureus.109923

**Published:** 2026-05-30

**Authors:** Smrutisree Mohapatra, Kunalsen Jagatdeo, Ankita Roy, Nishikanta Thatoi, Nishikanta Muduli, Basanti Kumari Pathi, Kumudini Panigrahi

**Affiliations:** 1 Microbiology, Kalinga Institute of Medical Sciences, Bhubaneswar, IND

**Keywords:** aspergillosis, fungal culture, galactomannan antigen, invasive fungal infection, koh mount

## Abstract

Background and objectives

Invasive fungal infections (IFIs) are the most prevalent potentially fatal opportunistic mycoses in immunocompromised individuals. Early detection is essential for treating these patients. Biomarkers, measured in blood or other clinical samples, are effective supplements to conventional pathologic and microbiologic diagnostic methods for IFIs. We conducted this study to determine the positivity of serum galactomannan (GM) in clinically diagnosed IFI. Also, we assessed the diagnostic power of GM for predicting invasive aspergillosis (IA), the most common cause of IFIs.

Methodology

This retrospective study analyzed clinically diagnosed patients with IFIs admitted to Kalinga Institute of Medical Sciences (KIMS), Bhubaneswar, India, from January 2023 to December 2025. The study included the blood samples received for serum GM antigen testing during the study period. We evaluated the diagnostic performance of the GM antigen. We also plotted the receiver operating characteristic (ROC) curve and determined the area under the curve (AUC).

Results

We analyzed data from 172 clinically diagnosed patients with IFIs. *Aspergillus flavus* consistently showed the highest GM levels, followed closely by *Aspergillus fumigatus* and *Aspergillus niger*. The test demonstrated high sensitivity (82.4%) but low specificity (46.8%). The AUC was 0.69. Differences in age, gender distribution, and year-over-year prevalence were statistically significant (*P* < 0.001).

Conclusions

GM antigen detection is useful for identifying IA. It demonstrated improved sensitivity and specificity compared with conventional methods (potassium hydroxide (KOH) and culture). Future multicenter studies incorporating polymerase chain reaction (PCR) and β-D-glucan are warranted to validate these findings.

## Introduction

Invasive fungal Infections (IFIs) are systemic infections in which fungi enter the body, replicate in tissues, causing direct tissue damage as well as inflammatory cascades [1,2]. Over 650 million individuals globally suffer from fungal infections each year; of these, over 7 million have severe IFIs, which cause over 1.35 million fatalities [3]. The use of broad-spectrum antibiotics, immunosuppressants, various catheters, surgery, prolonged hospital stays, glucocorticoids, and serious underlying illnesses such as organ transplantation and hematologic malignancies is strongly associated with the incidence of IFIs [4,5]. *Candida*, Mucorales, *Aspergillus*, *Cryptococcus*, and *Pneumocystis* species are the most frequent causal agents [6].

The incidence rates of invasive aspergillosis (IA), the most prevalent IFI, range from 5.5% to 20% [7,8]. The primary risk factors for IA are allogeneic stem cell transplantation and sustained severe neutropenia (neutrophils <500 cells/mL) with long-term corticosteroid use [9,10]. Early detection and intense treatment may improve the prognosis, but IA-related mortality ranges from 36% to 90% [11,12].

The most prevalent Aspergillus species that causes IA is *Aspergillus fumigatus*. In untreated or complicated cases, *A. fumigatus* may cause hematogenously disseminated fungal infection by spreading from the lungs to other organs [13].

The clinical signs of IFIs are frequently challenging to differentiate from those caused by viral or bacterial illnesses. It is still difficult to diagnose IFI in patients with impaired immune systems. Furthermore, the standard conventional culture procedures have shortcomings and lack sensitivity in diagnosing IFIs. Non-culture-based methods (PCR-based DNA detection or fungal serological biomarker) in blood or respiratory samples are crucial supplementary tests [14,15].

Fungal serological tests include tools that detect antigens of fungal pathogens or antibodies formed by the host to the corresponding invading pathogen [16]. These tests can be used as screening tools, as standalone diagnostics, or in combination with culture and microscopy to confirm a fungal infection [16-18]. However, variables like the test's timing in relation to antifungal therapy or the course of the infection can affect test sensitivity, potentially producing false-positive or false-negative results [17]. 

The cell walls of *Aspergillus *species contain a polymer called galactomannan (GM). The Aspergillus galactomannan antigen titer (AGT) test detects this polysaccharide [19]. Antigen testing is more specific in patients with compromised immunity because, even in cases of IA, there is frequently no Aspergillus GM antibody response [20]. Serum and bronchoalveolar lavage fluid versions of the AGT test are commercially available. The physician can select the specimen to use for diagnosis based on the patient's circumstances. Because serum is typically an easily available test, AGT analysis has been demonstrated to greatly enhance the identification of fungal infections in individuals with suspected IFIs [21]. The creation of a straightforward, indirect test for IA detection has sparked intense global debate.

According to studies, the circulating GM can be found five to eight days before aspergillosis manifests clinically [22]. The EB-A2 rat monoclonal antibody is used in the enzyme-linked immunosorbent assay (ELISA) (Platelia test) to detect GM [22]. Results are reported using an optical density index (ODI) [23]. To strengthen the test's specificity without changing its sensitivity, the threshold for serum positivity has been raised to 1 in recent years. The test's sensitivity can reach 71%, and its specificity can approach 90% with an ODI cutoff of 1 [24].

Given the high mortality associated with IA, there is an ongoing need to refine noninvasive, rapid, and reliable diagnostic methods. In this context, the present study was carried out to determine the positivity of GM antigen in serum of clinically diagnosed patients with IFIs. We also assessed the diagnostic power of GM in predicting IA.

## Materials and methods

In this retrospective study, we analyzed patients with clinically diagnosed IFIs admitted to Kalinga Institute of Medical Sciences (KIMS), Bhubaneswar, Odisha, between January 2023 and December 2025. The Institutional Ethics Committee of KIMS granted ethical clearance for the study (reference number: KIIT/KIMS/IEC/2617/2026). Because the study was retrospective, informed consent was not required.

Study procedure

Demographic and laboratory data of the participants were retrieved retrospectively from hospital records and laboratory registers. Demographic data included age and gender. Laboratory data included (serum GM level and interpretation (positive/negative), fungal culture findings). These data were compiled into a structured electronic database for analysis.

As part of routine clinical practices, blood samples from clinically suspected cases were processed in the Microbiology laboratory for IFI detection using serology. The patients were closely monitored throughout their hospital stay to identify sinopulmonary signs and symptoms. Commercially available ELISA kits were used to detect this antigen.

Aspergillus GM antigen was detected in patient serum using an enzyme-linked immunosorbent assay. Serum samples were processed using the Platelia Aspergillus Galactomannan EIA kit (Bio-Rad, France) in accordance with the manufacturer’s instructions. For each sample, 300 µL of test serum was mixed with 100 µL of treatment solution, vortexed, and heated at 120°C for 6 minutes in a dry heat block. Samples were then centrifuged at 10,000 rpm for 10 minutes. Approximately 50 µL of supernatant was mixed with 50 µL of conjugate in microtiter wells pre-coated with the monoclonal antibody EBA-2. After incubation at 37°C for 90 minutes, the plates were washed, and 200 µL of tetramethylbenzidine (TMB) substrate was added. The plates were incubated in the dark for 30 minutes, after which 100 µL of stopping solution was added to halt the reaction. Absorbance was measured at 450 nm with a reference wavelength of 620 nm. Results were expressed as an optical density (OD) index (ODI) relative to internal controls.

Each run included appropriate positive and negative controls. The index of each test sample was determined by dividing the sample's OD by the mean cutoff control OD; the findings were interpreted as index (I). The mean cutoff control OD was obtained by adding the OD values for each cutoff control and dividing the result by 2. For both male and female patients, serum samples with an index ≥1 and <1 were regarded as positive and negative for GM antigen, respectively [25].

Statistical analysis

This retrospective study employed convenience sampling. Sensitivity, specificity, positive predictive value (PPV), negative predictive value (NPV), accuracy, and 95% confidence intervals were computed to evaluate the serum GM performance. Analysis of variance (ANOVA) was used to evaluate quantitative variables among more than two groups with normally distributed data. The relationships between qualitative variables were evaluated using the chi-square test (χ²). Fisher’s exact test was applied whenever any expected cell count was fewer than five. Data analysis and plot creation were performed using R software version 4.3.2 (R Foundation for Statistical Computing, Vienna, Austria). *P*-values < 0.05 were considered statistically significant [26].

## Results

We analyzed data from 172 clinically diagnosed patients with IFIs. The mean age was 46.6 ± 14.2 years. The study population consisted of 98 males (57%) and 74 females (43%). Forty-nine (28.5%) cases were infected with *A. flavus*, followed by 41 (23.8%) cases with *A. fumigatus* and 35 (20.3%) with *A. niger*. The study spanned three years: 2023 (51 participants), 2024 (68 participants), and 2025 (53 participants). Differences in age, gender distribution, and year-over-year prevalence were statistically significant (*P* < 0.001) (Table 1).

**Table 1:** Demographic data for the study population (n). Continuous data are shown as the mean and standard deviation (SD). Categorical data are shown as numbers and percentages. Continuous data were analyzed using analysis of variance (ANOVA), and *F*-values were calculated. Categorical data were analyzed using the chi-square test, and chi-square values were calculated. A *P*-value < 0.05 was considered statistically significant.

Parameter	Total (*n* = 172)	*Aspergillus flavus *(*n* = 49)	*Aspergillus fumigatus* (*n *= 41)	*Aspergillus niger* (*n* = 35)	No growth (*n *= 47)	Statistical test used	Test statistics	*P*-value
Age (in years)	46.6 ± 14.2	45.2 ± 13.7	48.1 ± 11.6	47.4 ± 12.9	44.8 ± 11.5	ANOVA	10.781	<0.001
Gender, *n* (%)								
Male	98 (57%)	30 (61%)	27 (66%)	20 (57%)	21 (45%)	Chi-square test	23.413	<0.001
Female	74 (43%)	19 (39%)	14 (34%)	15 (43%)	26 (55%)			
Year, *n* (%)								
2023	51 (30%)	14 (28%)	11 (27%)	6 (17%)	20 (43%)	Chi-square test	34.508	<0.001
2024	68 (39%)	18 (37%)	18 (44%)	16 (46%)	16 (34%)			
2025	53 (31%)	17 (35%)	12 (29%)	13 (37%)	11 (23%)			

**Figure 1:**
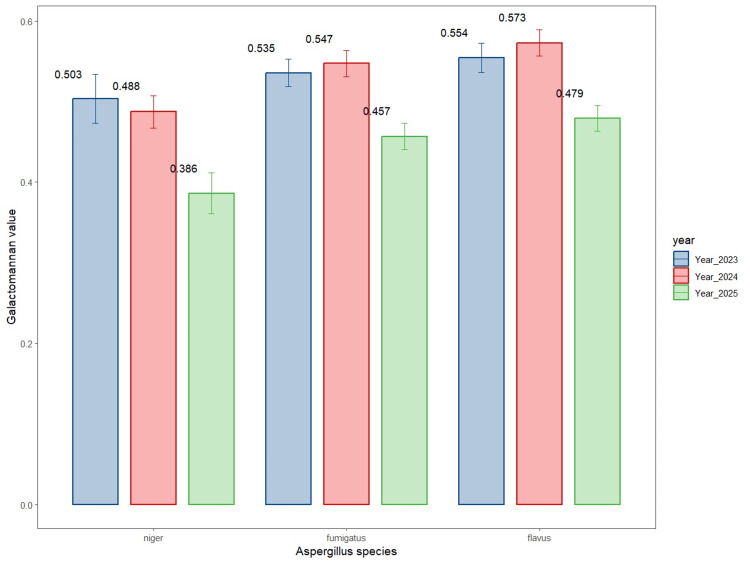
Mean galactomannan values of Aspergillus isolates. The bar plot shows galactomannan values for different *Aspergillus *species.

In Figure 1, the bar plots show the mean GM values for different *Aspergillus *species and their fluctuations over the three years (2023-2025). The error bars show the standard deviations of the means. The mean values are indicated above the corresponding bars. Data from the three years are shown in different colors. *A. flavus* consistently shows the highest GM levels, followed closely by *A. fumigatus*. *A. niger* maintains the lowest mean level among the identified species. Across all three species, there is a distinct pattern in which values either increase slightly or remain stable between 2023 and 2024, followed by a sharp decline in 2025. *A. flavus* reaches a peak mean of 0.573 in 2024 before dropping to 0.479 in 2025. *A. fumigatus* peaks at 0.547 in 2024, dropping to 0.457 in 2025. *A. niger* starts at 0.503 in 2023 and falls to its lowest point of 0.386 in 2025. The GM test is more likely to yield higher readings for *A. flavus* and *A. fumigatus* infections than for *A. niger*.​​​​​​

Table 2 tracks the mean GM values across different species and years. The overall mean GM value was 0.490. *A. flavus* exhibited the highest mean GM value at 0.535, followed by *A. fumigatus* with a mean of 0.518 and *A. niger* with a lower mean of 0.453. For almost every category, GM values peaked in 2024 and were lowest in 2025.

**Table 2:** Mean galactomannan values of the study population (n) analyzed from 2023 to 2025. The standard deviation (SD) and standard error (SE) of the mean galactomannan values were calculated. The data were analyzed using analysis of variance (ANOVA), and F-values were calculated. A *P*-value < 0.05 was considered statistically significant.

Parameter	Mean	SD	SE	Test statistics	*P*-value
Total population (*n *= 172)	0.490	0.091	0.001	23.413	<0.001
Year 2023 (*n *= 51)	0.506	0.079	0.003		
Year 2024 (*n *= 68)	0.516	0.093	0.003		
Year 2025 (*n *= 53)	0.440	0.081	0.003		
*Aspergillus flavus *(*n *= 49)	0.535	0.079	0.003	23.413	<0.001
Year 2023 (*n* = 14)	0.554	0.068	0.010		
Year 2024 (*n *= 18)	0.573	0.070	0.008		
Year 2025 (*n *= 17)	0.479	0.066	0.008		
*Aspergillus fumigatus* (*n *= 41)	0.518	0.073	0.004	23.413	<0.001
Year 2023 (*n *= 11)	0.535	0.057	0.010		
Year 2024 (*n *= 18)	0.547	0.069	0.008		
Year 2025 (*n *= 12)	0.457	0.056	0.009		
*Aspergillus niger* (*n *= 35)	0.453	0.097	0.006	23.413	<0.001
Year 2023 (*n *= 6)	0.503	0.074	0.025		
Year 2024 (*n *= 16)	0.488	0.080	0.010		
Year 2025 (*n *= 13)	0.386	0.091	0.014		
No growth (*n *= 47)	0.446	0.085	0.004	23.413	<0.001
Year 2023 (*n *= 20)	0.456	0.073	0.007		
Year 2024 (*n *= 16)	0.447	0.103	0.013		
Year 2025 (*n *= 11)	0.425	0.082	0.015		

**Figure 2:**
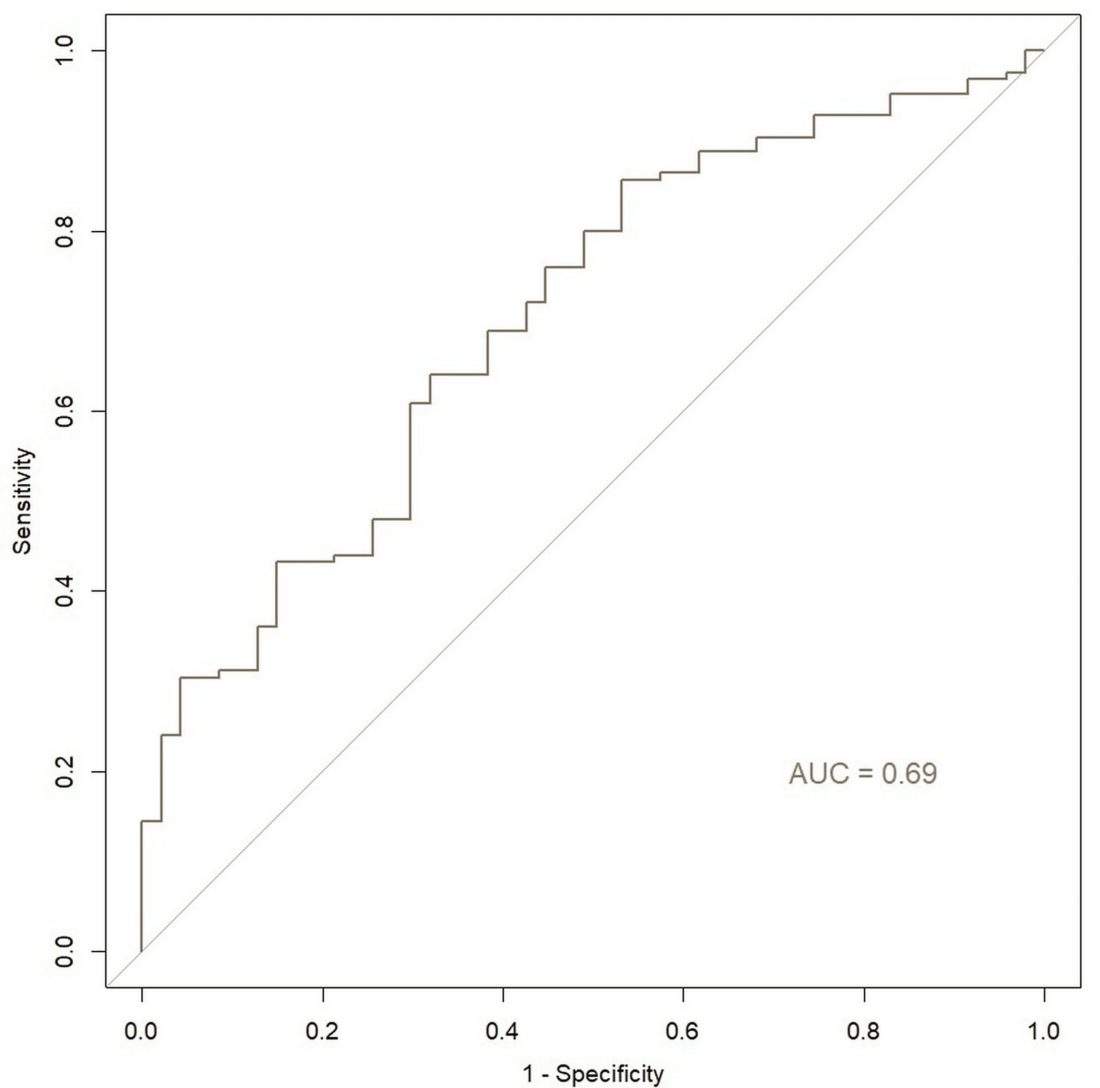
Receiver operating characteristic curve for galactomannan. The *x*-axis and *y*-axis represent specificity and sensitivity, respectively. AUC, area under the curve

In Figure 2, the plot shows sensitivity and 1-specificity on the *y*-axis and *x*-axis, respectively. The AUC was 0.69, suggesting that the test has a 69% chance of correctly distinguishing patients with IA from those without the disease. The curve shows high sensitivity but at the cost of specificity. This aligns with the data showing a high sensitivity of 82.4% but a low specificity of 46.8%. Since the brown stepped line is above the diagonal, the GM test is a significantly better diagnostic tool. The AUC of 0.69 and low specificity (46.8%) indicate that the test is an excellent screening tool.

Table 3 compares GM test results (positive/negative) with the actual presence of IA.

**Table 3:** Contingency table for galactomannan values. We calculated true-positive (TP), false-positive (FP), true-negative (TN), and false-negative (FN) values for galactomannan in assessing invasive aspergillosis.

Parameters	Invasive aspergillosis	No infection	Total
Galactomannan-positive	TP = 103	FP = 25	128
Galactomannan-negative	FN = 22	TN = 22	44
Total	125	47	172

Table 4 evaluates the GM test’s effectiveness as a diagnostic tool. The test is strong at correctly identifying those with the infection, with a sensitivity of 82.4%. The test is relatively weak at correctly identifying those without infection, with a specificity of 46.8%. The positive predictive value is 80.5%, indicating the highest likelihood of having IA. The negative predictive value is 50%, indicating a 50% chance of true IA clearance. The overall diagnostic accuracy of the GM test in this study was 72.7%.

**Table 4:** Statistical measures of galactomannan values and their 95% confidence intervals (CIs).

Statistics	Value	95% CI
Sensitivity	82.4%	74.6%-88.6%
Specificity	46.8%	32.1%-61.9%
Positive likelihood ratio	1.55	1.17-2.05
Negative likelihood ratio	0.38	0.23-0.61
Disease prevalence	72.7%	65.4%-79.2%
Positive predictive value	80.5%	75.7%-84.5%
Negative predictive value	50.0%	38.1%-61.9%
Diagnostic accuracy	72.7%	65.4%-79.2%

## Discussion

Patients with weakened immune systems are particularly vulnerable to IA. The use of biomarkers is necessary to improve the detection of fungal pathogens due to current diagnostic constraints. Cases are identified based on a positive culture for aspergillosis because most patients usually do not undergo invasive histological testing. Sensitive assays are, therefore, required for a precise diagnosis of the fungal infection [27,28].

The retrospective study presents a three-year overview of the role of serum GM in clinically diagnosed IFIs at a tertiary care hospital. In our study, the overall mean age was 46.6 ± 14.2 years, which is similar to the findings of Taghizadeh-Armaki et al., who reported a mean age of 46 ± 15.4 years [29]. Forty-nine (28.5%) cases were infected with *A. flavus*, followed by 41 (23.8%) cases with *A. fumigatus* and 35 (20.3%) with *A. niger*, which contradicts the findings of Namuwenge et al., who reported *A. flavus* in 7 (26.9%) cases, *A. niger* in 7 (26.9%) cases, and *A. fumigatus* in 3 (11.5%) cases [30].

The underlying disease, age, and assay cutoff values all affect the GM test's accuracy. The GM antigen sensitivity and specificity in our study were 82.4% and 46.8%, respectively, which can be compared to the findings of Hu et al., in which the sensitivity was 84.1%, and the specificity was 75.68%, which may be a useful tool in conjunction with clinical risk factors to guide diagnosis and treatment [31]. In our study, 103 test results were true positives (TPs), and 22 were false negatives (FNs), which were much higher than those reported by Hu et al., in which there were 25 TPs and 14 FNs [31].

The negative predictive value was 92%-98%, and the positive predictive value was 25%-62% in a study by Sarrafzadeh et al., which differed from our findings, in which the positive predictive value was 75.7%-84.5%, and the negative predictive value ranged from 38.1% to 61.9% [32]. The AUC in our study was 0.69, which was close to the findings of Dichtl et al., in which the AUC was 0.76 [33].

We advise routine GM antigen testing for each patient suspected of having a fungal infection because it is a reliable, quick, inexpensive test. In patients who also meet host characteristics and clinical criteria, a positive GM antigen test result may support a diagnosis of likely fungal infection and prompt the doctor to initiate empirical antifungal therapy. In patients with chronic neutropenic fever without radiologic or clinical symptoms, negative GM antigen assay results are more useful for confirming the absence of IA and for preventing empirical anti-mold therapy.

Limitations

The limitations of this study are discussed. The study is weaker because it is retrospective. Single-center designs may limit generalizability, as they may not be broadly applicable across populations and geographic regions. There was lower statistical power since the serum GM subgroup was small (*n* = 172). Clinical judgment was used to allocate patients to serum testing, which could introduce selection bias. Because studies with positive results are more likely to be published, potential publication bias may lead to an overestimation of diagnostic accuracy. We excluded 1,3-β-D-glucan testing and PCR assays, which could have improved diagnostic performance and enabled more thorough comparisons. Variations in reagent batches, individual patient differences, and test timing can affect the sensitivity and specificity of detection methods, leading to fluctuations in findings. Interpretation is further complicated by heterogeneity in cutoffs and by preceding antifungal therapy, as reported by Marr et al. [34]. Additionally, the development of IA and its identification may be affected by factors not accounted for in the study, such as patients’ concurrent drug use and specific immunological states.

## Conclusions

Serum GM antigen detection is a helpful and reliable diagnostic test for IFIs. For the diagnosis of IA in serum, the GM assay frequently offers great specificity, enabling early, focused antifungal treatment. The ability to distinguish invasive infection from colonization or non-infectious cases is greatly improved by incorporating GM testing into clinical protocols, particularly in immunocompromised patients. To improve the generalizability and reliability of the results, the limitations should be addressed in future studies by increasing the sample size and conducting investigations across multiple medical facilities in various locations.

An improved understanding of the developmental process and risk factors for IFIs would result from longitudinal patient tracking. Developing more precise and sensitive biomarker detection techniques for IFI is expected to increase diagnostic precision.
